# Fission yeast mitochondria are distributed by dynamic microtubules in a motor-independent manner

**DOI:** 10.1038/srep11023

**Published:** 2015-06-05

**Authors:** Tianpeng Li, Fan Zheng, Martin Cheung, Fengsong Wang, Chuanhai Fu

**Affiliations:** 1 Department of Biochemistry; 2 Department of Anatomy, The University of Hong Kong, Pokfulam, Hong Kong, China; 3 HKU-Shenzhen Institute of Research and Innovation, The University of Hong Kong, Shenzhen, China; 4 School of Life Sciences, Anhui Medical University, Hefei, Anhui 230032, China

## Abstract

The cytoskeleton plays a critical role in regulating mitochondria distribution. Similar to axonal mitochondria, the fission yeast mitochondria are distributed by the microtubule cytoskeleton, but this is regulated by a motor-independent mechanism depending on the microtubule associated protein mmb1p as the absence of mmb1p causes mitochondria aggregation. In this study, using a series of chimeric proteins to control the subcellular localization and motility of mitochondria, we show that a chimeric molecule containing a microtubule binding domain and the mitochondria outer membrane protein tom22p can restore the normal interconnected mitochondria network in mmb1-deletion (mmb1∆) cells. In contrast, increasing the motility of mitochondria by using a chimeric molecule containing a kinesin motor domain and tom22p cannot rescue mitochondria aggregation defects in mmb1∆ cells. Intriguingly a chimeric molecule carrying an actin binding domain and tom22p results in mitochondria associated with actin filaments at the actomyosin ring during mitosis, leading to cytokinesis defects. These findings suggest that the passive motor-independent microtubule-based mechanism is the major contributor to mitochondria distribution in wild type fission yeast cells. Hence, we establish that attachment to microtubules, but not kinesin-dependent movement and the actin cytoskeleton, is required and crucial for proper mitochondria distribution in fission yeast.

In general, mitochondria are organized in an interconnected tubular network within the cell. Proper mitochondria distribution is important for regulating metabolism, calcium homeostasis, neuronal functions, and cell proliferation. Although mitochondria are found to physically interact with a number of cytoplasmic organelles such as Endoplasmic Reticulum^1^,^2^, it is the cytoskeleton that plays a critical role in determining the patterns of mitochondria distribution in a cell-type dependent or an organism-specific manner. Generally, in plants and budding yeast, mitochondria are positioned by actin filaments^3^,^4^,^5^, whereas in many higher eukaryotic cells and fission yeast, mitochondria are distributed by microtubules. Intriguingly, in neuronal cells, short-range mitochondria movements and anchoring depend on actin filaments^6^,^7^, but long-range mitochondria movements and mitochondria distribution primarily depend on motor proteins and the microtubule cytoskeleton^8^,^9^,^10^.

For proper inheritance, the fission yeast mitochondria must be properly distributed in the cytoplasm by microtubules, depending on the microtubule associated protein (MAP) mmb1p^9^. In the absence of mmb1p, mitochondria lose the characteristic tubular network structure and aggregate within the cytoplasm, causing defects in mitochondria inheritance. It has been reported that the conventional kinesin-1 klp3p is not involved in regulating mitochondria distribution in fission yeast^11^. Therefore, fission yeast likely employs a passive motor-independent microtubule-based mechanism to regulate mitochondria positioning and thus serves as an excellent model organism for dissection of the various regulatory mechanisms underlying mitochondria distribution. Whether the motor-independent microtubule-based mechanism is sufficient for mitochondria distribution in fission yeast remains unclear.

In this study, we have engineered and employed a series of chimeric proteins, capable of targeting mitochondria to microtubules or to actin filaments and transporting mitochondria along microtubules, respectively, to dissect the various mechanisms in regulating mitochondria distribution in fission yeast. Targeting mitochondria to microtubules almost completely restores, if not entirely, the normal mitochondria interconnected network in mmb1-deletion (mmb1∆) cells. In contrast, forcing mitochondria to associate with actin filaments does not rescue the mitochondria aggregation phenotype in mmb1∆ cells, and instead, these mitochondria mainly concentrate at the actomyosin ring, causing cytokinesis defects. In addition, we further show that motile mitochondria result in their aggregation preferentially at one cell end. Thus, this study demonstrates that the passive motor-independent microtubule-based mechanism is the major contributor to mitochondria distribution in fission yeast.

## Results

### A chimeric protein containing the microtubule and mitochondria binding domains is sufficient for proper positioning of mitochondria in the cytoplasm

**Figure 1:**
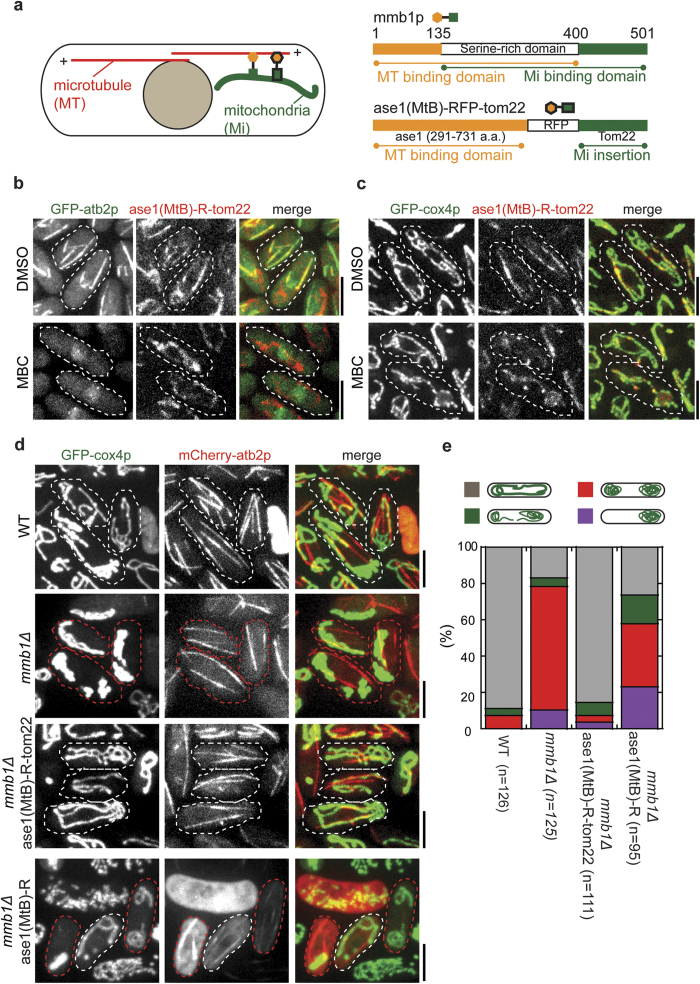
A chimera of ase1p microtubule binding domain and tom22p can rescue the mitochondria aggregation phenotype in mmb1∆ cells. (**a**) Schematic diagram depicting the subcellular localization of mmb1p and the chimera ase1(MtB)-TagRFP-tom22. Domain structures of mmb1p and the chimera ase1p(MtB)-TagRFP-tom22 are illustrated. (**b**) Maximum projection images of wild type cells expressing GFP-atb2p (microtubule marker) and ase1p(MtB)-TagRFP-tom22 (from an ase1p promoter). The cells were treated either with DMSO or with the microtubule depolymerizing drug MBC for 10 min before imaging. Ase1p(MtB)-TagRFP-tom22 colocalized with microtubules, and in the presence of MBC, microtubules were depolymerized and ase1p(MtB)-TagRFP-tom22 exhibited amorphous localization in the cytoplasm. Scale bar, 5 μm. (**c**) Maximum projection images of wild type cells expressing GFP-cox4 (mitochondria marker) and ase1p(MtB)-TagRFP-tom22 in the absence or presence of MBC. Ase1p(MtB)-TagRFP-tom22 colocalized with mitochondria in the cytoplasm. Scale bar, 5 μm. (**d**) Maximum projection images of wild type (WT), mmb1-deletion (mmb1∆), mmb1∆ ase1p(MtB)-TagRFP-tom22, and mmb1∆ ase1p(MtB)-TagRFP cells expressing GFP-cox4 and mCherry-atb2. Mitochondria in wild type and mmb1∆ ase1p(MtB)-TagRFP-tom22 cells were tubular (white dash lines) whereas mitochondria in mmb1∆ and mmb1∆ ase1p(MtB)-TagRFP cells displayed aggregation (red dash lines). Scale bar, 5 μm. (**e**) Quantification of the indicated patterns of mitochondria distribution in WT, mmb1∆, mmb1∆ ase1p(MtB)-TagRFP-tom22, and mmb1∆ ase1p(MtB)-TagRFP cells. Note that ase1p(MtB)-TagRFP-tom22 but not ase1p(MtB)-TagRFP can rescue mitochondria aggregation defects in mmb1∆ cells.

Mmb1p tethers mitochondria to microtubules via its C-terminal mitochondria binding domain and the N-terminal microtubule binding region (Fig. 1A)^9^. We then asked whether these two regions are sufficient for mediating proper mitochondria positioning. This prompted us to engineer a chimeric protein (designed as ase1(MtB)-RFP-tom22), in which a RFP coding region is flanked by the previously reported ase1p microtubule binding domain (a.a. 297–731) at its N-terminus and the mitochondria outer membrane protein tom22p at its C-terminus (Fig. 1a)^12^,^13^,^14^. Note that a similar chimeric molecule containing tom22p has been shown to be able to target mitochondria to microtubules in neuronal cells^15^. As control, we first confirmed the subcellular localization of the fusion proteins RFP-tom22p and ase1(MtB)-RFP (Supplementary Fig. S1a). Confocal imaging showed that RFP-tom22p was localized to mitochondria (Supplementary Fig. S1b). In addition, ectopic expression of RFP-tom22p did not result in defects in mitochondria distribution in wild-type (WT) cells, nor did it rescue mitochondria aggregation in mmb1∆ cells (Supplementary Fig. S1c). Consistent with previous studies^14^, ase1(MtB)-RFP was localized to microtubules without affecting mitochondria distribution (Supplementary Fig. S1d). Intriguingly, ase1(MtB)-RFP-tom22 was found to colocalize with microtubules and mitochondria in the cytoplasm (Figs 1b,c). The localization of ase1(MtB)-RFP-tom22 to mitochondria was further evidenced by complete colocalization of ase1(MtB)-RFP-tom22 with the mitochondria marker GFP-cox4p in the absence of microtubules (Fig. 1c). Therefore, these results suggest that ase1(MtB)-RFP-tom22 can bind both microtubules and mitochondria.

To assess the effect of ase1-RFP-tom22 on mitochondria distribution, we quantified the patterns of mitochondria distribution in WT and mmb1∆ cells, and mmb1∆ cells overexpressing ase1(MtB)-RFP-tom22 or ase1(MtB)-RFP (Figs 1d,e). In agreement with our previous report^9^, mmb1∆ cells exhibited massive mitochondria aggregation either at one cell end or at both ends. In contrast, expression of ase1(MtB)-RFP-tom22, not ase1(MtB)-RFP and RFP-tom22 (See supplementary Fig. 1), in mmb1∆ cells significantly increased the percentage of cells with wild-type tubular mitochondria distribution (Figs 1d,e). Thus, we conclude that an mmb1p-like molecule capable of binding mitochondria and microtubules can efficiently mediate proper mitochondria distribution.

### The chimeric kinesin protein klp3(MDo)-RFP-tom22 can transport mitochondria along the microtubule lattice

**Figure 2:**
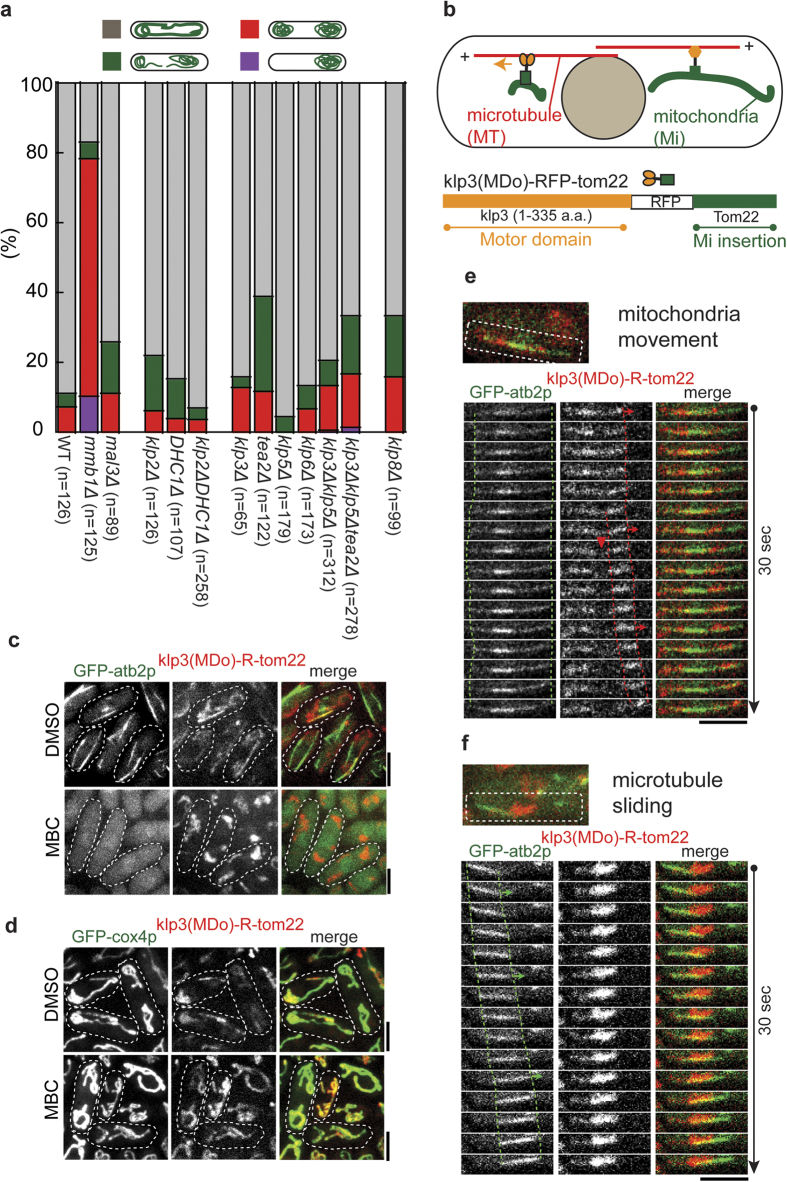
A chimera of klp3 motor domain and tom22p can drive mitochondria movement *in vivo*. (**a**) Quantification of the patterns of mitochondria distribution in the indicated cells. Compared to mmb1∆ cells, none of the motor-deletion mutants, either alone or in combination, caused significant mitochondria aggregation defects. (**b**) Schematic diagram depicting the subcellular localization of klp3(MDo)-TagRFP-tom22. The motor domain (a.a. 1–335) of klp3p is fused to TagRFP tagged tom22 at its N-terminus. (**c**) Maximum projection images of wild type cells expressing GFP-atb2p and klp3p(MDo)-TagRFP-tom22 (from an ase1p promoter). Cells were treated either with DMSO or with MBC for 10 min before imaging. Klp3p(MDo)-TagRFP-tom22 colocalized with microtubules and exhibited amorphous cytoplasmic localization upon microtubule depolymerization with MBC. Scale bar, 5 μm. (**d**) Maximum projection images of wild type cells expressing GFP-cox4 and klp3p(MDo)-TagRFP-tom22. Klp3p(MDo)-TagRFP-tom22 colocalized with mitochondria in the cytoplasm. Scale bar, 5 μm. (**e**) Maximum projection time-lapse images of a cell expressing GFP-atb2p and klp3p(MDo)-TagRFP-tom22. A divided mitochondrion (red arrowhead) marked by klp3p(MDo)-TagRFP-tom22 rapidly moved along the microtubule lattice towards the cell tip (red arrows). Green and red dash lines mark microtubule and mitochondria tips, respectively. Montage images were constructed using the region indicated in the analyzed cell. Scale bar, 5 μm. (**f**) A microtubule fragment (green dash lines) was slid away from the cell tip and towards the cell center (green arrows) by a stationary mitochondrion. Montage images were constructed using the region indicated in the analyzed cell. Scale bar, 5 μm

Although both neurons and fission yeast utilize microtubules to distribute mitochondria, they accomplish this by apparently different approaches. In axons, an active mechanism is employed, with kinesins and dynein responsible for anterograde and retrograde transport of mitochondria, respectively^10^. However, in the fission yeast cells, it appears that mitochondria are distributed passively through attachment to dynamic microtubules^9^. It has been implicated that the conventional kinesin-1 klp3p has no role in mitochondria distribution in fission yeast^11^. However, there are six kinesins (i.e. klp2p^16^, klp3p^11^, tea2p^17^, klp5p^18^,^19^, klp6p^18^,^19^, klp8p^20^) and one dynein^21^ present in the cytoplasm of fission yeast, and therefore they may be potential players in mitochondria distribution. Among these motor proteins, klp2p and dynein are minus-end-directed while klp3p, tea2p, klp5p, klp6p, and klp8p are plus-end-directed motor proteins. To test if these motor proteins are involved in regulating mitochondria distribution, our first attempt was to systematically examine the patterns of mitochondria distribution in single kinesin- and dynein-deletion mutant strains. As shown in Fig. 2a, only klp3∆, tea2∆ and klp8∆ mutant cells showed a very small degree of mitochondria aggregation, comparable to mal3∆ cells the absence of which has been known to result in short microtubules, thus indirectly causing mitochondria aggregation^22^. These data extended previous findings that kinesins and dynein did not play a major and direct role in fission yeast mitochondria distribution. To reinforce this conclusion, functional redundancy of the motor proteins should be considered. As the motor proteins have multiple roles in regulating mitosis and meiosis, it remains a technical challenge to knock out all six kinesins and dynein to assess the effect of combined deletions on mitochondria distribution. Instead, we sought to simultaneously knock out motor proteins with the same directionality. Since klp8p is localized to the actomyosin ring at the cell cortex^20^, it is unlikely that klp8p is involved in mitochondria distribution. In addition, klp5p and klp6p function as a heterodimer^19^, and it is therefore conceivable that deletion of either one would lead to identical phenotypes. As shown in Fig. 2a, the double and triple deletion mutants displayed comparable patterns of mitochondria distribution to the single deletion mutant. Hence, we conclude that kinesins and dynein play a negligible role in mitochondria distribution in fission yeast.

### Kinesin-driven mitochondria movement causes mitochondria aggregation in fission yeast

We then sought to investigate further why an active kinesin-dependent mechanism is not beneficial for mitochondria distribution in fission yeast. To this end, we created a chimeric molecule containing the kinesin-1 klp3p motor domain and tom22p (designated as klp3(MDo)-RFP-tom22) (Fig. 2b). As expected, similar to ase1(MtB)-RFP-tom22, klp3(MDo)-RFP-tom22 colocalized with microtubules (Fig. 2c) and mitochondria (Fig. 2d), suggesting that it can target mitochondria to microtubules. Next, we employed high temporal live-cell imaging to examine the dynamics of klp3(MDo)-RFP-tom22 and microtubules. In many cases, the tubular klp3(MDo)-RFP-tom22 signals (also represent mitochondria as klp3(MDo)-RFP-tom22 colocalized with mitochondria) were observed to split apart into small fragments and move rapidly towards cell tips along the microtubule lattice (Fig. 2e). Such rapid mitochondria movement was not seen in wild type cells. Thus, we conclude that klp3(MDo)-RFP-tom22 can drive mitochondria movement *in vivo*. Occasionally, microtubule fragments were observed to be slid through a stationary mitochondrion towards the cell center, further confirming that klp3(MDo)-RFP-tom22 has motor activity capable of microtubule sliding (Fig. 2f). Hence, klp3(MDo)-RFP-tom22 is a functional chimeric kinesin capable of transporting mitochondria.

**Figure 3:**
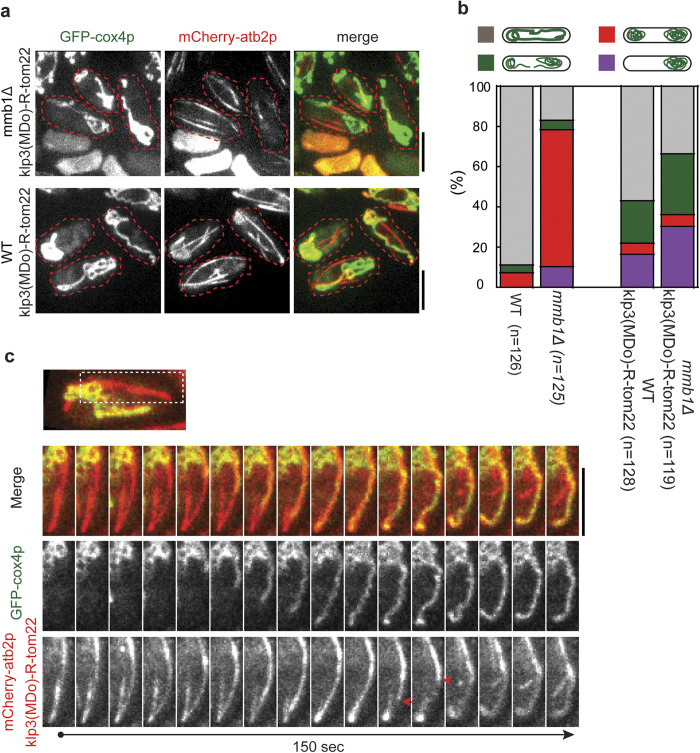
The klp3p(MDo)-TagRFP-tom22 chimera partially rescues mitochondria aggregation caused by the absence of mmb1p. (**a**) Maximum projection images of WT klp3p(MDo)-TagRFP-tom22 and mmb1∆ klp3p(MDo)-TagRFP-tom22 cells expressing mCherry-atb2p and GFP-cox4p. Indicated cells (red dash lines) displayed mitochondria aggregation. Scale bar, 5 μm.(**b**) Quantification of the indicated patterns of mitochondria distribution in WT, mmb1∆, WT klp3(MDo)-TagRFP-tom22 cells, and mmb1∆ klp3(MDo)-TagRFP-tom22 cells. Note that klp3(MDo)-TagRFP-tom22 partially rescued mitochondria aggregation in mmb1∆ cells but caused mitochondria aggregation in WT cells.(**c**) Maximum projection time-lapse images of an mmb1∆ klp3(MDo)-TagRFP-tom22 cell expressing mCherry-atb2p and GFP-cox4p. A tubular mitochondrion was stretched from the aggregate and extended along the microtubule lattice to the cell tip within 30 sec. Red arrows mark the depolymerizing microtubule plus end. Montage images were constructed using the region indicated in the analyzed cell. Scale bar, 5 μm.

Next, we assessed the effect of klp3(MDo)-RFP-tom22 on mitochondria distribution. Interestingly, expression of klp3(MDo)-RFP-tom22 doubled the number of the mmb1∆ cells with normal tubular mitochondria distribution (i.e. complete rescue) and also significantly increased the number of the mmb1∆ cells that showed mitochondria aggregation at both cell ends but with minor connection (i.e. partial rescue) (Figs 3a,b). Time-lapse imaging indicated that the complete or partial rescue may be attributed to the motor activity of klp3(MDo)-RFP-tom22 which occasionally extended a mitochondria tubule from aggregates and carried it along microtubules towards cell tips (Fig. 3c). However, klp3(MDo)-RFP-tom22 also tripled the number of the mmb1∆ cells that exhibited mitochondria aggregation at only one cell end (30% and 10% for the klp3(MDo)-RFP-tom22 mmb1∆ cells and mmb1∆ cells, respectively) (Fig. 3b). Therefore, expression of klp3(MDo)-RFP-tom22 could triple the risk for an mmb1∆ cell to lose mitochondria completely after cell division. Strikingly, even in the presence of mmb1p, klp3(MDo)-RFP-tom22 expression caused mitochondria aggregation at one cell end in ~16% cells (vs 0% wild type cells) (Figs 3a,b). As control, we also examined the subcellular localization of GFP tagged klp3 motor domain alone (GFP-klp3(MDo)) and found that majority of GFP-klp3(MDo) were resided in the cytoplasm and its microtubule localization (at the spindle) became apparent only when cells enter mitosis (Supplementary Fig. S2a-c). Ectopic expression of GFP-klp3(MDo) did not cause significant mitochondria aggregation in WT cells, nor did it rescue or exacerbate mitochondria aggregation in mmb1∆ cells (Supplementary Fig. S2d). Thus, these findings clearly argue that unlike axonal mitochondria, motile mitochondria in fission yeast, if driven by kinesins, may affect proper mitochondria distribution.

### Targeting mitochondria to actin filaments leads to cytokinesis defects in fission yeast

**Figure 4:**
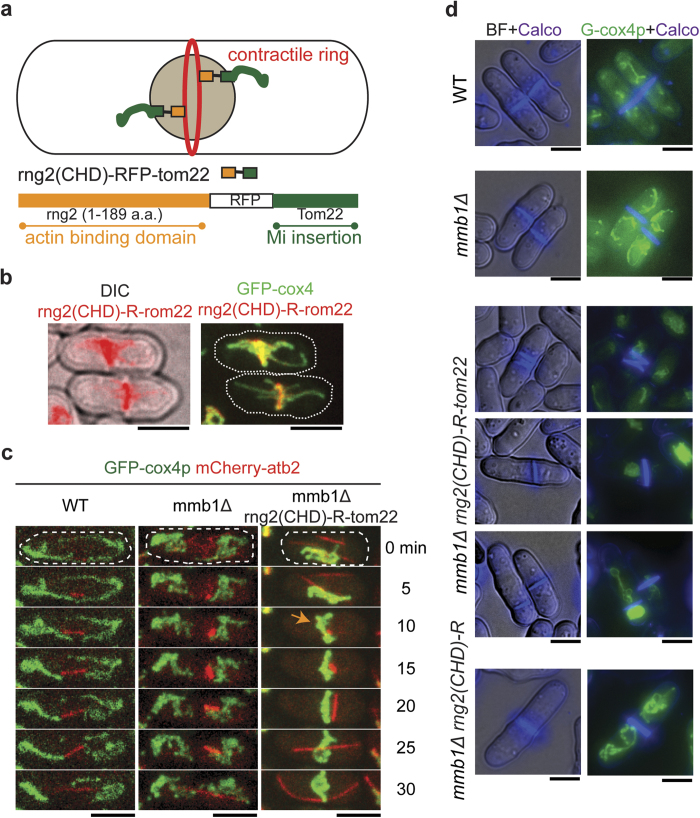
A chimera of rng2 actin binding domain and tom22p localizes to the actomyosin ring and causes cytokinesis defects. (**a**) Schematic diagram depicting the subcellular localization and the domain structure of the chimera rng2(CHD)-TagRFP-tom22. (**b**) Maximum projection images of cells expressing GFP-cox4 and rng2(CHD)-TagRFP-tom22 (from an ase1p promoter). Rng2(CHD)-TagRFP-tom22 and mitochondria colocalized at the actomyosin ring, with tubular mitochondria projected towards the cell tips. Scale bar, 5 μm.(**c**) Maximum projection time-lapse images of WT, mmb1∆, and mmb1∆ rng2(CHD)-TagRFP-tom22 cells expressing mCherry-atb2 and GFP-cox4. Mitochondria (yellow arrow) were forced to position at the actomyosin ring by rng2(CHD)-TagRFP-tom22 whereas mitochondria in WT and mmb1∆ cells did not localize to the cell center. Scale bar, 5 μm.(**d**) Calcofluor-white staining of WT, mmb1∆, mmb1∆ rng2(CHD)-TagRFP-tom22, and mmb1∆ rng2(CHD)-TagRFP cells. Note that only *mmb1∆* rng2(CHD)-TagRFP-tom22 cells displayed defects in septum formation and positioning. Scale bar, 5 μm.

In contrast to the microtubule-based regulatory mechanisms for mitochondria distribution in fission yeast, plants and budding yeast, depends on actin filaments instead of microtubules, to distribute mitochondria^4^. To further elucidate why fission yeast cells do not employ the actin-based mechanism for mitochondria distribution and inheritance, we sought to target mitochondria to actin filaments by taking advantage of a previously defined actin binding domain rng2(CHD)^23^ which was tagged with RFP (RFP-rng2 (CHD)) and displayed localization to actin filaments (Supplementary Fig. S3a and S3b). Ectopic expression of TagRFP-rng2(CHD) did not cause noticeable changes in mitochondria morphology, distribution, and separation (Supplementary Fig. S3b and S3c). We then fused rng2(CHD) to tom22p (designated as rng2(CHD)-RFP-tom22) for targeting mitochondria to actin filaments (Figs 4a,b). However, in rng2(CHD)-RFP-tom22 expressing strain, instead of localizing to the actin cables and patches at cell tips, mitochondria was predominantly associated with the actomyosin ring in the cell center (Figs 4b,c). Similar to mmb1∆ cells, the mitochondria mispositioning leaded to spindle misorientation during mitosis in rng2(CHD)-RFP-tom22 expressing cells (Fig. 4c). This finding is consistent with the previous report that spindle alignment relies on proper attachment of mitochondria to the spindle pole body^24^. The preferential localization of mitochondria at the septum region prompted us to test whether the mitochondria mispositioning could cause cytokinesis defects. Indeed, calcofluor white staining showed remarkable cytokinesis defects including off-center positioning and multiple septa in mmb1∆ cells expressing rng2(CHD)-RFP-tom22 but not in mmb1∆ cells expressing rng2(CHD)-RFP and in WT and mmb1∆ cells (Fig. 4d). Taken together, these results suggest that artificially targeting mitochondria to the actomyosin ring cause cytokinesis defects. Hence, if driven by actin filaments, mitochondria distribution may affect faithful cell division in fission yeast.

## Discussion

Mitochondria must be properly distributed within a cell for inheritance, generally depending on the microtubule cytoskeleton and/or actin filaments. Here, using a synthetic biology approach, we demonstrate that a chimeric protein containing mitochondria and microtubule binding domains can replace mmb1p to distribute mitochondria in fission yeast whereas motile mitochondria and targeting mitochondria to actin filaments impairs mitochondria distribution and causes cytokinesis defects, respectively. Collectively, this work establishes that binding stationary mitochondria to dynamic microtubules is necessary and sufficient for the formation of the characteristic tubular mitochondria network in fission yeast.

Motile axonal mitochondria are important for neuronal functions^15^,^25^. It is equally important to dock stationary mitochondria within axons for maintaining local ATP homeostasis^15^. Unlike axonal mitochondria that employ both active and passive microtubule-based mechanisms for mitochondria distribution, the fission yeast mitochondria are stationary and are passively distributed by dynamic microtubules^8^,^9^ (Fig. 1). Question remains of why an active transport mechanism does not involve in mitochondria distribution in fission yeast. It is likely due to the typical small size of the cell, approximate 14 μm in length. This length is much shorter than the length of the axon of a typical neuron. Considering the velocity of the conventional kinesin-1 at ~1 μm/sec^26^, it takes only ~7 seconds for a mitochondrion to reach to the cell tips in fission yeast if a kinesin-dependent mechanism was involved. Furthermore, microtubules in fission yeast form 2–3 antiparallel bundles within the cytoplasm, with their plus ends pointing towards the cell tips and generally contacting the cell tips for approximate 1 minute before undergoing catastrophe^27^,^28^. Therefore, if mitochondria were transported by kinesins in fission yeast, many mitochondria are expected to accumulate to cell tips within the 1 minute window before microtubule depolymerization occurs. Consistently, we observed that a mitochondrial tubular tip can be positioned from the cell center to the cell tip within 20–30 seconds by the chimeric kinesin klp3(MDo)-RFP-tom22 (Fig. 3c). As a result, ~16% of the wild type cells ectopically expressing klp3(MDo)-RFP-tom22 exhibit the one-end mitochondria aggregation phenotype (Fig. 3b), and consequently, these defective cells may asymmetrically partition mitochondria to two daughter cells after cell division, impairing cell growth. On the other hand, motile mitochondria may alter microtubule organization and dynamics (Fig. 2f), thus affecting essential microtubule-based functions such as cell polarity regulation^29^. Therefore, due to the small cell size and the special microtubule organization and dynamics, fission yeast has evolved a passive microtubule-based but motor-independent mechanism for mitochondria distribution. It is likely that this mechanism requires only a protein that contains both microtubule and mitochondria binding domains (Fig. 1).

Actin filaments are responsible for mitochondria distribution in plants and the budding yeast *S.cerevisiae*^4^. Interestingly, such mechanism does not operate in fission yeast. In fission yeast, actin filaments are organized as cables and patches at cell tips in interphase and as a compact ring structure during mitosis^29^. It is therefore impossible for the actin filaments to evenly distribute mitochondria within the cytoplasm. Interestingly, rng2(CHD)-RFP-tom22 targets mitochondria mainly to the actomyosin ring, not to the cell tip region. This mitochondria localization pattern could be due to the different dynamic properties between the actomyosin ring and the actin cables and patches. The actomyosin ring is compact and relatively stable, whereas the actin cables and patches at cell tips are dynamic. Therefore, the chimera rng2(CHD)-RFP-tom22 may stably attach mitochondria to the actomyosin ring instead of the dynamic actin cables and patches. In addition, localization of mitochondria to the actomyosin ring appears to severely affect assembly and positioning of the actomyosin ring as multiple septa and mis-positioned septa are frequently observed in cells expressing rng2(CHD)-RFP-tom22 (Fig. 4d). Thus, due to the subcellular localization restrictions, actin filaments may not be suitable for mediating even mitochondria distribution in the cytoplasm.

In plants, microtubules are confined to the cell cortex, organized into a parallel array, whereas actin filaments are more complex and dynamic, forming a meshwork structure in the cytoplasm^30^,^31^. Therefore, the cytoplasmic actin meshwork structure may be more suitable to evenly distribute mitochondria, compared to the membrane bound microtubules. Similarly, the interphase budding yeast cells have actin filaments that are enriched at the budding sites where few dynamic microtubules reside^32^. This special spatial organization of budding yeast actin filaments allow mitochondria to be efficiently delivered to the daughter cells through the budding sites. Hence, depending on the cytoskeleton architecture, different organisms have evolved specific mechanisms for mitochondria distribution and inheritance.

As the protein domains used in our chimeric molecules are derived from the evolutionarily conserved proteins ase1p (PRC1 in human), klp3p (kinsin-1), rng2p (IQGAP in human), and tom22p (the TOM complex subunit Tom22), these engineered molecules may also be functional in other eukaryotic cells, and therefore, they may also serve as excellent tools for dissecting mitochondria dynamics in higher eukaryotic cells.

## Methods

### Yeast strains and plasmids

Random spore digestion or tetra-dissection approaches were employed to create yeast strains, as previously described^33^. All culture media were purchased from Formedium (www.formedium.com). For creating the plasmids ase1(MtB)-RFP-tom22, klp3(MDo)-RFP-tom22, and rng2(CHD)-RFP-tom22, the inserts ase1(a.a. 291–731), klp3(a.a. 1–335), rng2(a.a. 1–189) and tom22 were first ligated to pET28a-TagRFP (TagRFP was inserted at the BamH1 site of pET28a) at the sites BglII and NcoI, and BamHI and NotI, respectively, and the intermediate fragments ase1(MtB)-RFP-tom22, klp3(MDo)-RFP-tom22, and rng2(CHD)-RFP-tom22 were then ligated to pJK148 or pJK210 at the sites BglII and NotI. These new plasmids were linearized and integrated either at the leu1.32 locus or at the ura4-294 locus and were expressed from the ase1 promoter. All yeast strains and plasmids used in this study are listed in Supplementary Table S1 and S2, respectively.

### Microscopy and data analysis

We followed the previous description for imaging with a Perkin Elmer spinning-disk confocal microscope equipped with a Zeiss PlanApo 100X/1.4 NA objective and a Photometrics EMCCD camera Evolve 512^34^. All imaging were carried out at 26 °C in a temperature controllable incubator. For maximum projection analysis, Z-stack images consisting of 21 planes with a step size of 0.25 μm were acquired. For high temporal resolution analysis, Z-stack images consisting of 3 planes with a step size of 0.5 μm were acquired every 10 sec. MBC treatment assays were carried as previously described^9^. Detailed imaging conditions are described in the supplement. Images were analyzed with Metamorph (www.moleculardevices.com), and graphs were generated with Kaleidagraph 4.5 (www.synergy.com).

## Additional Information

**How to cite this article**: Li, T. *et al*. Fission yeast mitochondria are distributed by dynamic microtubules in a motor-independent manner. *Sci. Rep*. **5**, 11023; doi: 10.1038/srep11023 (2015).

## Supplementary Material

Supplementary Information
