# Supplementary material for: Fission yeast mitochondria are distributed by dynamic microtubules in a motor-independent manner
Source: Sci Rep. 2015 Jun 5;5:11023. doi: 10.1038/srep11023 (PMC4457142; doi:10.1038/srep11023)

Fission yeast mitochondria are distributed by dynamic microtubules in a motor-independent manner

Tianpeng Li[1,3], Fan Zheng[1,3], Martin Cheung[2], Fengsong Wang[4], Chuanhai Fu[1,3,*]

[1]Department of Biochemistry, [2]Department of Anatomy, The University of Hong Kong, Pokfulam, Hong Kong, China

[3]HKU-Shenzhen Institute of Research and Innovation, The University of Hong Kong, Shenzhen, China

[4]School of Life Sciences, Anhui Medical University, Hefei, Anhui 230032, China

*Correspondence should be addressed to C.F. (chuanhai@hku.hk) (Phone: (+852) 3917 9208; Fax: (+852) 2855 1254)

Supplementary Information includes

Supplementary figure legends S1-3

Supplementary imaging conditions

Supplementary tables S1 and S2

Supplementary figure S1-3

Supplementary figure legends

Figure S1: TagRFP tagged ase1p microtubule binding domain localizes to microtubules, and TagRFP tagged tom22p cannot rescue mitochondria aggregation defects in mmb1Δ cells.

(a) Schematic diagram depicting the subcellular localization of TagRFP tagged ase1p microtubule binding domain (ase1(MtB)-RFP) and TagRFP tagged tom22p (RFP-tom22). Domain structures of the two fusion proteins are illustrated.

(b) Maximum projection images of wild type cells expressing GFP-cox4p and TagRFP-tom22p. The cells were treated either with DMSO or with MBC for 10 min before imaging. TagRFP-tom22 colocalized with mitochondria in the absence or presence of microtubules. Scale bar, 5 μm.

(c) Quantification of the indicated patterns of mitochondria distribution in WT, WT TagRFP-tom22, mmb1Δ, and mmb1Δ TagRFP-tom22 cells. Note that TagRFP-tom22 did not cause mitochondria aggression in WT cells, nor did it rescue mitochondria aggregation defects in mmb1Δ cells.

(d) Maximum projection images of wild type cells expressing GFP-atb2p and ase1-TagRFP. The cells were treated either with DMSO or with MBC for 10 min before imaging. As expected, ase1-TagRFP localized to microtubules. Scale bar, 5 μm.

(e) Quantification of the indicated patterns of mitochondria distribution in WT, WT ase1(MtB)-TagRFP, mmb1Δ, and mmb1Δ ase1(MtB)-TagRFP cells. Note that ase1(MtB)-TagRFP did not cause mitochondria aggression in WT cells, nor did it rescue mitochondria aggregation defects in mmb1Δ cells.

Figure S2: Ectopic expression of TagRFP tagged tom22p has no effect on mitochondria aggregation.

(a) Schematic diagram depicting the subcellular localization of TagRFP tagged klp3 motor domain (GFP-klp3(MDo)).

(b) Maximum projection images of wild type cells expressing RFP-cox4p and GFP-klp3(MDo). Note that GFP-klp3(MDo) was localized in the cytoplasm and its localization to microtubules became apparent during mitosis (the spindle marked by the green arrowhead). Scale bar, 5 μm.

(c) Maximum projection images of wild type cells expressing mCherry-atb2p and GFP-klp3(MDo). Note that GFP-klp3(MDo) was localized in the cytoplasm and its localization to microtubules became apparent during mitosis (the spindle marked by the green arrowhead). Scale bar, 5 μm.

(d) Quantification of the indicated patterns of mitochondria distribution in WT, WT GFP-klp3(MDo), mmb1Δ, and mmb1Δ GFP-klp3(MDo) cells. Note that GFP-klp3(MDo) did

not rescue mitochondria aggregation defects in mmb1Δ cells, nor did it cause apparent mitochondria aggression in WT cells.

Figure S3: TagRFP tagged rng2 actin binding domain is localized to actin filaments.

(a) Schematic diagram depicting the subcellular localization of TagRFP tagged rng2 actin binding domain (rng2(CHD)-RFP).

(b) Maximum projection images of wild type cells expressing GFP-cox4p and rng2(CHD)-RFP. Note that rng2(CHD)-RFP was localized to the actomyosin ring in mitosis and to actin patches and cables in interphase. Ectopic expression of rng2(CHD)-RFP had no effect on mitochondria distribution. Scale bar, 5 μm.

(c) Maximum projection time-lapse images of mmb1Δ rng2(CHD)-TagRFP-tom22 and mmb1Δ rng2(CHD)-TagRFP cells expressing mCherry-atb2 and GFP-cox4. Mitochondria (yellow arrow) were forced to position at the actomyosin ring by rng2(CHD)-TagRFP-tom22 but not rng2(CHD)-TagRFP. Scale bar, 5 μm.

<u>Supplementary Imaging conditions</u>

Figure 1b-d and Figure S1b & S1d, Maximum projection Z-stack images: 21 optical planes, 0.25 μm step size, 500 ms mCherry exposure, 500 ms GFP exposure, 3X EM gain.

Figure 2c & 2d and Figure S2b & S2c, Maximum projection Z-stack images: 21 optical planes, 0.25 μm step size, 500 ms mCherry exposure, 500 ms GFP exposure, 3X EM gain.

Figure 2e & 2f, live-cell single-plane images: 1 optical plane, 2 Sec intervals, 500 ms mCherry exposure, 500 ms GFP exposure, 3X EM gain.

Figure 3a, Maximum projection Z-stack images: 21 optical planes, 0.25 μm step size, 500 ms mCherry exposure, 500 ms GFP exposure, 3X EM gain.

Figure 3c, Maximum projection live-cell Z-stack images: 3 optical plane, 10 Sec intervals, 500 ms mCherry exposure, 500 ms GFP exposure, 3X EM gain.

Figure 4b-d and Figure S3b, Maximum projection Z-stack images: 21 optical planes, 0.25 μm step size, 500 ms mCherry exposure, 500 ms GFP exposure, 3X EM gain.

Figure 4c and Figure S3c, Maximum projection live-cell Z-stack images: 11 optical plane, 0.5 μm step size, 1 min intervals, 500 ms mCherry exposure, 500 ms GFP exposure, 3X EM gain.

Supplementary Tables

Table S1. Yeast strains

Strain	Genotype	Source
Figure 1		
CF.2172	GFP-atb2:KanR Leu1-32:ase1P*-ase1(291-731)-TagRFP-tom22 leu1-32 ura4-D18 h-	This study
CF.2310	nmt1P*-GFP-cox4:LEU+ Ura4-294:ase1P*-ase1(291-731)-TagRFP-tom22 ade6-m210? leu1-32 ura4-294 h-	This study
CF.2379	nmt1P*-GFP-cox4:LEU+ mCherry-atb2:HygR ade6-m210? leu1-32 ura4-294 h+	This study
CF.2256	mmb1Δ:KanR+ nmt1P*-GFP-cox4:LEU+ mCherry-atb2:HygR ade6-m210? leu1-32 ura4-294 h?	This study
CF.2480	mmb1Δ:KanR+ nmt1P*-GFP-cox4:LEU+ mCherry-atb2:HygR Ura4-294:ase1P*-ase1(291-731)-TagRFP-tom22 ade6-m210? leu1-32 ura4-294 h?	This study
CF.3066	nmt1P*-GFP-cox4:LEU+ mCherry-atb2:HygR Ura4-294:nmt1P*-ase1(291-731)-TagRFP ade6-m210? leu1-32 ura4-294 h+	This study
CF.3067	mmb1Δ:KanR+ nmt1P*-GFP-cox4:LEU+ mCherry-atb2:HygR [pSGP572a-nmt1P*-ase1(291-731)-TagRFP:URA4+] ade6-m210? leu1-32 ura4-294 h-	This study
Figure 2		
CF.2379	nmt1P*-GFP-cox4:LEU+ mCherry-atb2:HygR ade6-m210? leu1-32 ura4-294 h+	This study
CF.2256	mmb1Δ:KanR+ nmt1P*-GFP-cox4:LEU+ mCherry-atb2:HygR ade6-m210? leu1-32 ura4-294 h?	This study
CF.389	mal3Δ:KanR+ nmt1P*-GFP-cox4:LEU+ mCherry-atb2:HygR ade6-m210? leu1-32 ura4-D18 h+	This study
CF.379	klp2Δ:URA4+ nmt1P*-GFP-cox4:LEU+ mCherry-atb2:HygR ade6-m210? leu1-32 ura4-D18 h+	This study
CF.380	klp3Δ:KanR+ nmt1P*-GFP-cox4:LEU+ mCherry-atb2:HygR ade6-m210? leu1-32 ura4-D18 h+	This study
CF.381	tea2Δ:KanR+ nmt1P*-GFP-cox4:LEU+ mCherry-atb2:HygR ade6-m210? leu1-32 ura4-D18 h+	This study
CF.382	klp5Δ:URA4+ nmt1P*-GFP-cox4:LEU+ mCherry-atb2:HygR ade6-m210? leu1-32 ura4-D18 his3.D1? h-	This study
CF.383	klp6Δ:URA4+ nmt1P*-GFP-cox4:LEU+ mCherry-atb2:HygR ade6-m210? leu1-32 ura4-D18 his3.D1? h-	This study
CF.384	klp8Δ:KanR+ nmt1P*-GFP-cox4:LEU+ mCherry-atb2:HygR ade6-m210? leu1-32 ura4-D18 h-	This study
CF.384	DHC1Δ:KanR+ nmt1P*-GFP-cox4:LEU+ mCherry-atb2:HygR ade6-m210? leu1-32 ura4-D18 h+	This study
CF.3085	klp2Δ:URA4+ DHC1Δ:KanR+ nmt1P*-GFP-cox4:LEU+ mCherry-atb2:HygR ade6-m210? leu1-32 ura4-D18 h-	This study
CF.3060	klp3Δ:KanR+ klp5Δ:URA4+ nmt1P*-GFP-cox4:LEU+ mCherry-atb2:HygR ade6-m210? leu1-32 ura4-D18 his3D1? h+	This study
CF.3062	klp3Δ:KanR+ klp5Δ:URA4+ tea2Δ:KanR+ nmt1P*-GFP-cox4:LEU+ mCherry-atb2:HygR ade6-m210? leu1-32 ura4-D18 his3D1? h?	This study
CF.2173	GFP-atb2:KanR Leu1-32:ase1P*-klp3(1-335)-TagRFP-tom22 leu1-32 ura4-D18 h-	This study
CF.2311	nmt1P*-GFP-cox4:LEU+ Ura4-294:ase1P*-klp3(1-335)-TagRFP-tom22 ade6-m210? leu1-32 ura4-294 h-	This study
Figure 3		
CF.2481	mmb1Δ:KanR+ nmt1P*-GFP-cox4:LEU+ mCherry-atb2:HygR Ura4-	This study

	294:ase1P*-klp3(1-335)-TagRFP-tom22 ade6-m210? leu1-32 ura4-294 h?	
CF.2657	nmt1P*-GFP-cox4:LEU+ mCherry-atb2:HygR Ura4-294:ase1P*-klp3(1-335)-TagRFP-tom22 ade6-m210? leu1-32 ura4-294 h+	This study
Figure 4		
CF.2492	nmt1P*-GFP-cox4:LEU+ Ura4-294:ase1P*-rng2(1-189)-TagRFP-tom22 ade6-m210? leu1-32 ura4-294 h?	This study
CF.2379	nmt1P*-GFP-cox4:LEU+ mCherry-atb2:HygR ade6-m210? leu1-32 ura4-294 h+	This study
CF.2256	mmb1Δ:KanR+ nmt1P*-GFP-cox4:LEU+ mCherry-atb2:HygR ade6-m210? leu1-32 ura4-294 h?	This study
CF.2493	mmb1Δ:KanR+ nmt1P*-GFP-cox4:LEU+ mCherry-atb2:HygR Ura4-294:ase1P*-rng2(1-189)-TagRFP-tom22 ade6-m210? leu1-32 ura4-294 h?	This study
CF.3075	mmb1Δ:KanR+ nmt1P*-GFP-cox4:LEU+ mCherry-atb2:HygR [pSGP572a-nmt1P*- rng2(1-189)-TagRFP:URA4+] ade6-m210? leu1-32? ura4-294 h-	This study
Figure S1		
CF.3148	GFP-cox4:KanMX Leu1-32:ase1P*-TagRFP-tom22 ade6-m210 leu1-32 ura4-294 h-	This study
CF.3150	GFP-cox4:KanMX leu1-32:ase1P*-TagRFP-tom22 mCherry-atb2:HygR ade6-m210? leu1-32 ura4-294 h-	This study
CF.3151	mmb1Δ:KanR+ GFP-cox4:KanMX leu1-32:ase1P*-TagRFP-tom22 mCherry-atb2:HygR ade6-m210? leu1-32 ura4-294 h?	This study
CF.3065	GFP-atb2:KanMX ura4-294: ase1P*-ase1(291-731)-TagRFP leu1-32 ura4-294 h?	This study
Figure S2		
CF.3068	nmt1P*-RFP-cox4:LEU+ [pSGP573-nmt1P*-GFP-klp3(1-335):URA4+] ade6-m210 leu1-32 ura4-294 h-	This study
CF.3070	nmt1P*-RFP-cox4:LEU+ GFP-atb2:KanMX [pSGP573-nmt1P*-GFP-klp3(1-335):URA4+] ade6-m210 leu1-32 ura4-294 h+	This study
CF.3071	mmb1Δ:KanR+ nmt1P*-RFP-cox4:LEU+ GFP-atb2:KanMX [pSGP573-nmt1P*-GFP-klp3(1-335):URA4+] ade6-m210? leu1-32 ura4-294 h-	This study
Figure S3		
CF.2492	nmt1P*-GFP-cox4:LEU+ [pSGP572a- nmt1P*-rng2(1-189)-TagRFP:URA4+] ade6-m210? leu1-32 ura4-294 h?	This study

Table S2. Plasmids

Plasmid	Genotype	Source
Figure 1		
pCF.1693	pJK210- ase1P*-ase1(291-731)-TagRFP-tom22	This study
pCF.2022	pSGP572a- nmt1P*-ase1(291-731)-TagRFP	This study
Figure 2		
pCF.1676	pJK148- ase1P*-klp3(1-335)-TagRFP-tom22	This study
pCF.1694	pJK210- ase1P*-klp3(1-335)-TagRFP-tom22	This study
Figure 3		
pCF.1694	pJK210- ase1P*-klp3(1-335)-TagRFP-tom22	This study
Figure 4		
pCF.1828	pJK210- ase1P*-rng2(1-189)-TagRFP-tom22	This study
pCF.2024	pSGP572a- nmt1P*-rng2(1-189)-TagRFP	This study

Figure S1		
pCF.2017	pJK148- ase1P*-TagRFP-tom22	This study
Figure S2		
pCF.2026	pSGP573-nmt1P*-GFP-klp3(1-335)	This study

Supplementary Figure S1 Li et al.

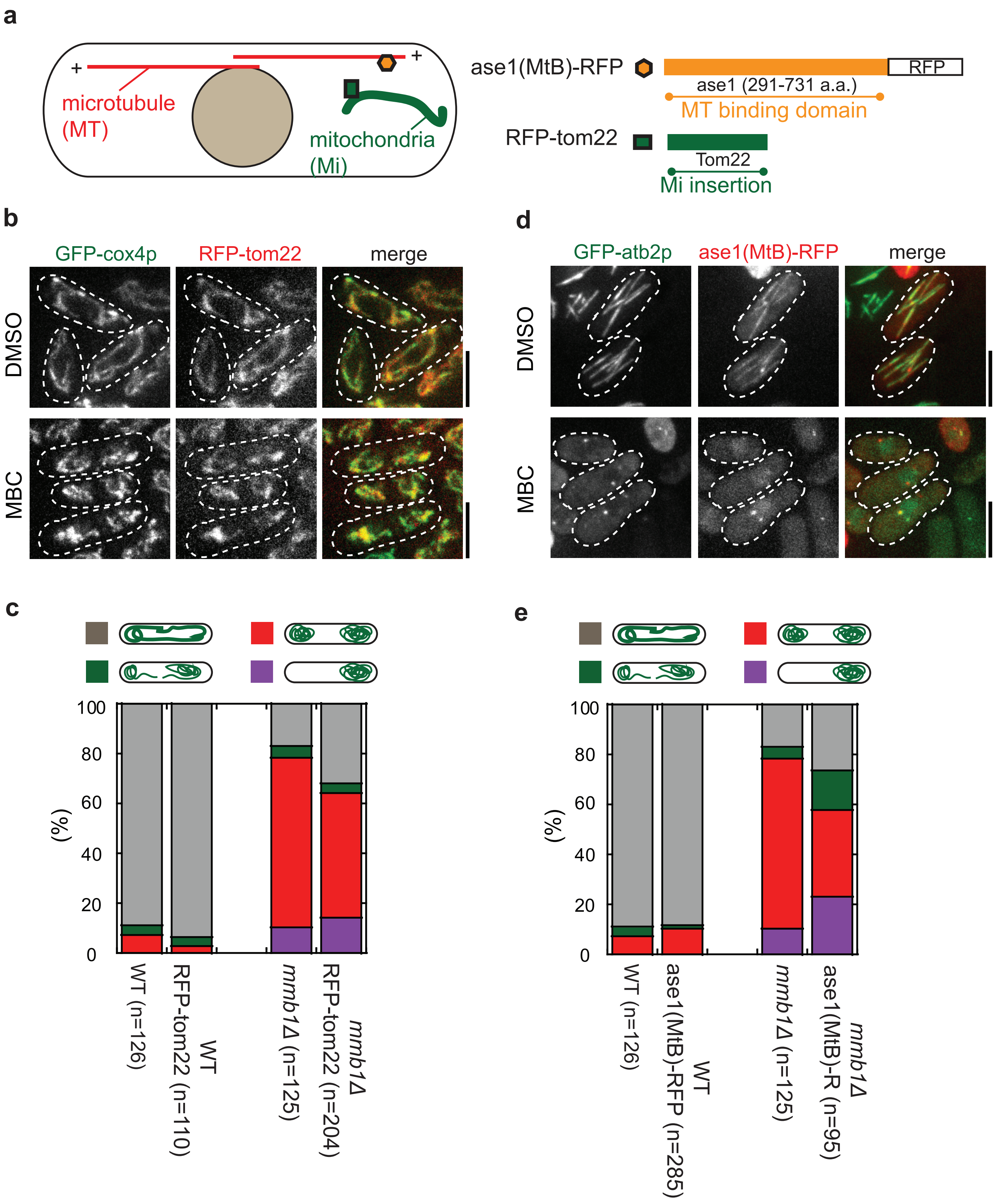

Supplementary Figure S2 Li et al.

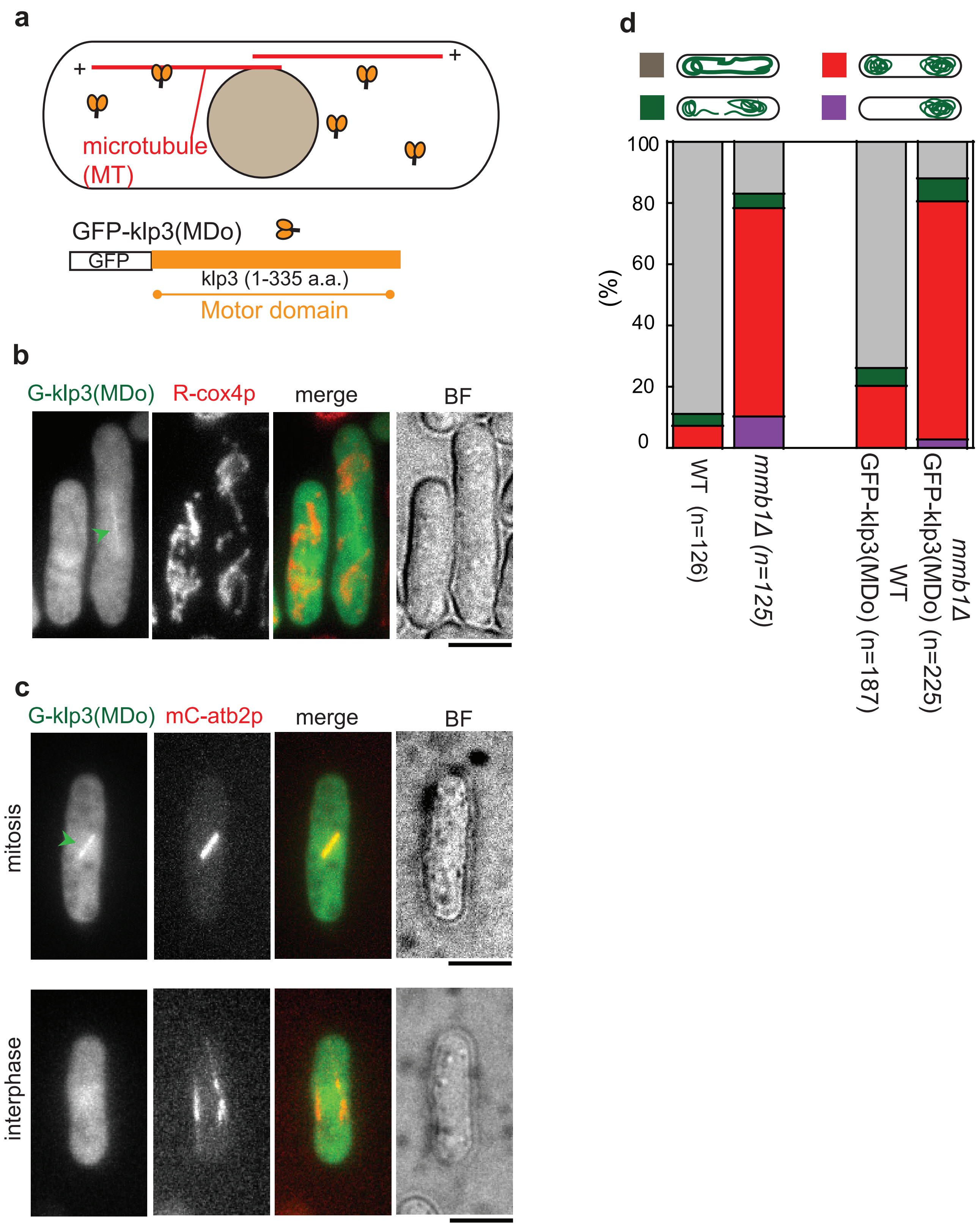

Supplementary Figure S3 Li et al.

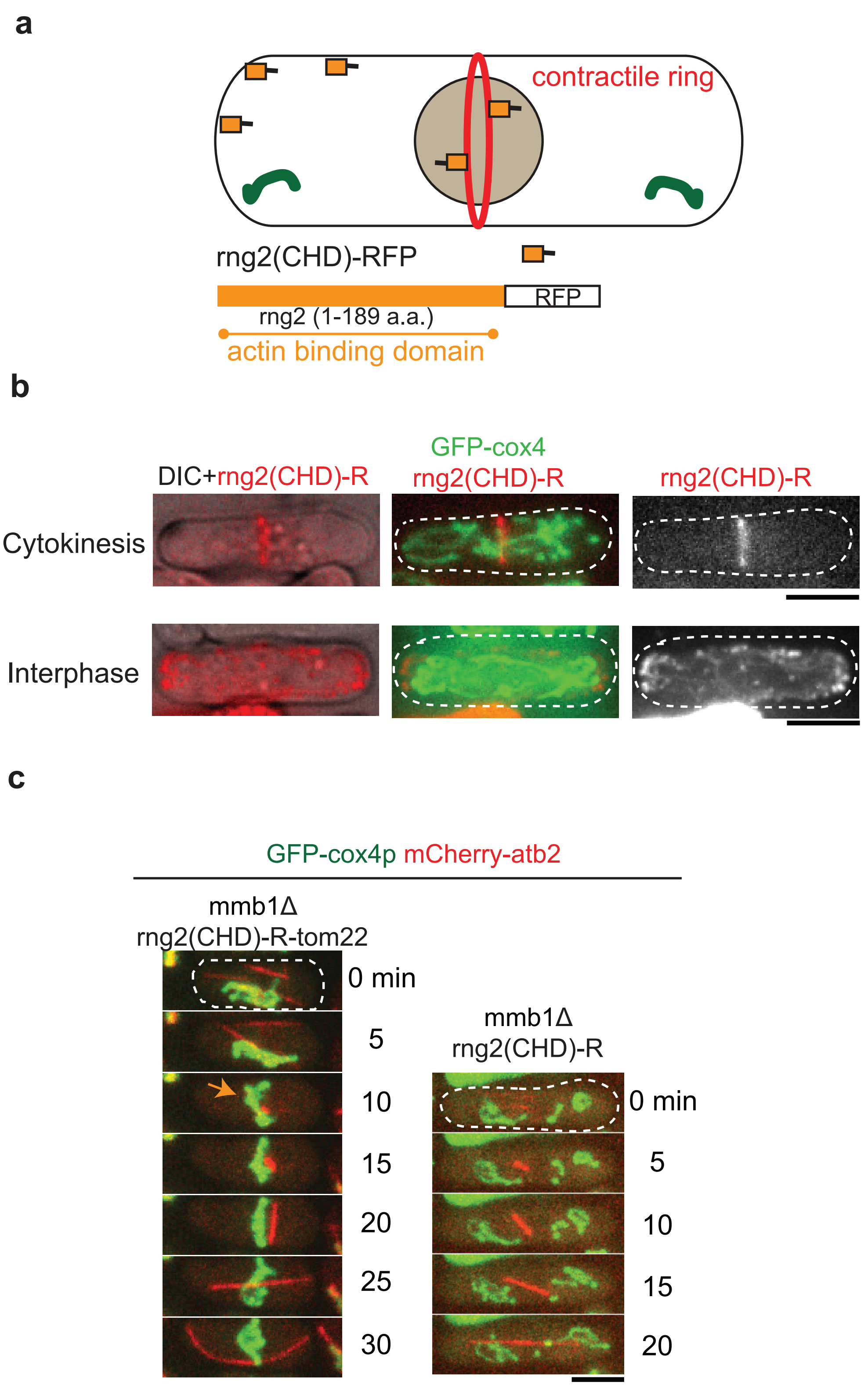

Supplement: Supplementary Information [file srep11023-s1.pdf]
